# Secondary Metabolites of the Rice Blast Fungus *Pyricularia oryzae*: Biosynthesis and Biological Function

**DOI:** 10.3390/ijms21228698

**Published:** 2020-11-18

**Authors:** Takayuki Motoyama

**Affiliations:** Chemical Biology Research Group, RIKEN CSRS, Wako, Saitama 351-0198, Japan; tmotoyam@riken.jp

**Keywords:** plant pathogenic fungus, *Magnaporthe oryzae*, secondary metabolite biosynthetic gene cluster, biological function

## Abstract

Plant pathogenic fungi produce a wide variety of secondary metabolites with unique and complex structures. However, most fungal secondary metabolism genes are poorly expressed under laboratory conditions. Moreover, the relationship between pathogenicity and secondary metabolites remains unclear. To activate silent gene clusters in fungi, successful approaches such as epigenetic control, promoter exchange, and heterologous expression have been reported. *Pyricularia oryzae*, a well-characterized plant pathogenic fungus, is the causal pathogen of rice blast disease. *P. oryzae* is also rich in secondary metabolism genes. However, biosynthetic genes for only four groups of secondary metabolites have been well characterized in this fungus. Biosynthetic genes for two of the four groups of secondary metabolites have been identified by activating secondary metabolism. This review focuses on the biosynthesis and roles of the four groups of secondary metabolites produced by *P. oryzae*. These secondary metabolites include melanin, a polyketide compound required for rice infection; pyriculols, phytotoxic polyketide compounds; nectriapyrones, antibacterial polyketide compounds produced mainly by symbiotic fungi including endophytes and plant pathogens; and tenuazonic acid, a well-known mycotoxin produced by various plant pathogenic fungi and biosynthesized by a unique NRPS-PKS enzyme.

## 1. Introduction

Filamentous fungi, including plant pathogenic fungi, produce a wide variety of secondary metabolites with unique and complex structures. However, the relationship between pathogenicity and secondary metabolites remains unclear in most cases. Filamentous fungi are a rich source of secondary metabolites for drug development. Whole-genome sequencing analyses have revealed that filamentous fungi possess many more secondary metabolism genes than expected, suggesting that most secondary metabolite biosynthetic genes are silent under laboratory conditions. To utilize fungal secondary metabolite production ability, secondary metabolism genes have been activated through many approaches, including epigenetic control, manipulation of global regulators, ribosome engineering, overexpression of pathway-specific transcription factors, co-culture, and heterologous expression of secondary metabolite gene clusters [1,2,3,4].

**Figure 1:**
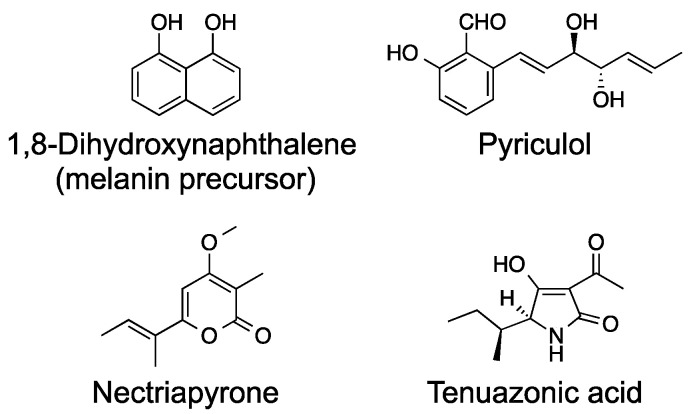
Chemical structures of secondary metabolites from the rice blast fungus *P. oryzae*.

*Pyricularia oryzae* (syn. *Magnaporthe oryzae*) is the causal pathogen of rice blast disease and is a well-characterized plant pathogen. *P. oryzae* infects rice plants through an infection-specific organ, the appressorium, and proliferates inside the rice plant via filamentous growth and causes rice blast disease [5]. *P. oryzae* is also rich in secondary metabolism genes and shown to have 22 polyketide synthase (PKS) genes and eight non-ribosomal peptide synthetase (NRPS) genes [6,7]. Biosynthetic genes for only four groups of secondary metabolites (melanin, pyriculols, nectriapyrones, and tenuazonic acid) have been well characterized in *P. oryzae* (Figure 1). Biosynthetic genes for two (nectryapyrones and tenuazonic acid) of the four groups of secondary metabolites have been identified by activating secondary metabolism.

Here, I review the biosynthesis and biological roles of secondary metabolites in the rice blast fungus *P. oryzae*. This review mainly focuses on the four groups of secondary metabolites shown in Figure 1.

## 2. Melanin

*P. oryzae* produces the black pigment melanin (Figure 1), which is essential for rice infection [8]. Melanin is not a toxin, but this secondary metabolite is essential for infection by the mechanism shown below. *P. oryzae* forms an infection-specific organ, appressorium, and infects rice plants through this organ. Appressorium formation and appressorium melanin formation are essential for rice infection. The invasion of rice plants is achieved by an infection peg that is formed at the base of an appressorium, which adheres tightly to the host surface. For successful penetration from the infection peg, mechanical force exerted by appressoria is necessary [8]. An appressorial melanin layer between the cell wall and cell membrane is required for the generation of the mechanical force. The turgor forces are focused toward the epidermal surfaces of the rice plant, and the pressure inside the appressoria has been estimated to be as high as 8 MPa [9,10]. This pressure can be produced by 3.2 M glycerol formed inside the appressorium [11]. Melanin was proposed to function as a semipermeable membrane that passes water but not glycerol and as a structural support for this very high pressure.

**Figure 2:**
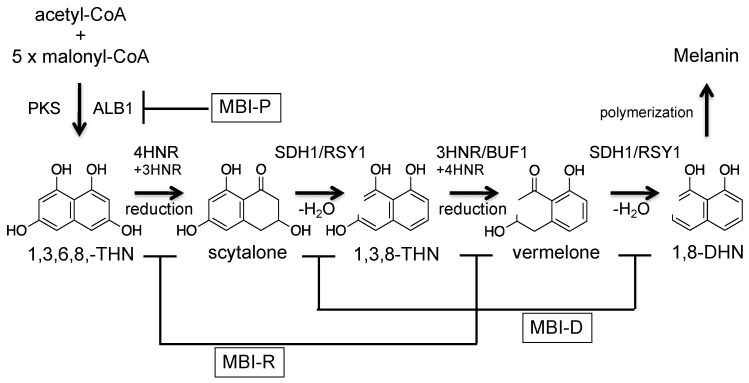
Melanin biosynthetic pathway of the rice blast fungus *P. oryzae*.

Melanin is a well-known black pigment of biological origin. One type of fungal melanin is dihydroxynaphthalene (DHN)-melanin, which is biosynthesized by polymerizing the polyketide compound 1,8-dihydroxynaphthalene (1,8-DHN) [12,13]. *P. oryzae* produces DHN-melanin and biosynthetic genes have been identified, and the biosynthetic pathway has been elucidated (Figure 2) [14,15,16,17]. The PKS enzyme ALB1/MGG_07219 biosynthesizes the backbone compound 1,3,6,8-tetrahydroxynaphthalene (1,3,6,8-THN). Melanin was originally proposed as a pentaketide compound. However, from the analysis of an ALB1 homolog in a closely related fungus, *Colletotrichum lagenarium*, it has been shown that melanin is a hexaketide compound and the backbone (1,3,6,8-THN) is biosynthesized using an acetyl-CoA and five malonyl-CoA [18]. Then, 1,3,6,8-THN is converted to 1,8-DHN by using three enzymes: 1,3,6,8-THN reductase (4HNR), scytalone dehydratase (SDH1/RSY1), and 1,3,8-trihydroxynaphthalene (1,3,8-THN) reductase (3HNR/BUF1). Finally, 1,8-DHN is polymerized to form DHN-melanin. Melanin biosynthesis can be induced by epigenetic control [19].

Melanin biosynthetic enzymes are targets of agrochemical development, and three types of commercial melanin biosynthesis inhibitors (MBIs) have been developed (Figure 2). These inhibitors are classified into three groups: MBI-R (tricyclazole, pyroquilon, and phthalide), MBI-D (carpropamid, diclocymet, and fenoxanil), and MBI-P (tolprocarb) [20,21,22]. The targets of MBI-R, MBI-D, and MBI-P are 1,3,8-trihydroxynaphthalene reductase (3HNR/BUF1), scytalone dehydratase (SDH1/RSY1), and polyketide synthase (ALB1), respectively. MBIs are environmentally friendly agrochemicals because MBIs inhibit fungal infection without inhibiting fungal growth.

## 3. Pyriculols

**Figure 3:**
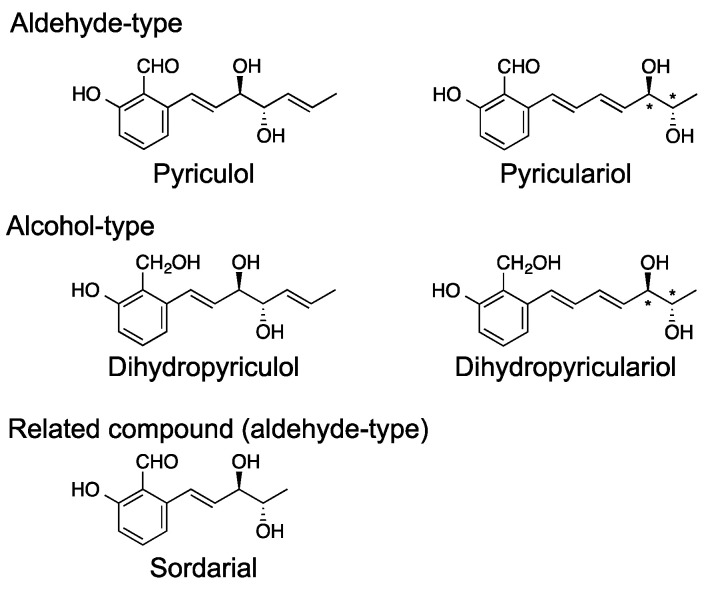
Structures of the pyriculols and a related compound.

Pyriculol (Figure 1) is a well-known secondary metabolite of the rice blast fungus and is known as a phytotoxin [23]. Several analogs of pyriculol have been reported. The main analogs are dihydropyriculol [24], pyriculariol [25], and dihydropyriculariol [26] (Figure 3). It is also shown that griseaketides, analogs of pyriculol, are produced by an isolate of rice blast fungus [27]. Pyriculols are classified into two groups: Alcohol-type (dihydropyriculol and dihydropyriculariol) and aldehyde-type (pyriculol and pyriculariol). It has been shown that aldehyde derivatives induce lesion-like necrosis on rice leaves, while alcohol derivatives are inactive [24,26,28]. Four analogs are produced simultaneously [28] and interconversion between oxidized aldehyde analogs and reduced alcohol derivatives is expected. Currently, it is not clear why and how *P. oryzae* produces both alcohol and aldehyde analogs. Identification of the genes responsible for this oxidoreductive conversion will help to answer this question.

Pyriculols are polyketide compounds, and the biosynthetic gene cluster has been recently identified [28]. The PKS gene (*MGG_10912*/*MoPKS19*) and other genes predicted to be responsible for the biosynthesis of pyriculols have been identified [28]. It has been suggested that aldehyde-type analogs are produced first and converted to alcohol-type analogs by the reduction reaction [26]. The gene (*MGG_10961*/*MoC19OXR1*) responsible for the oxidation of alcohol-type analogs to aldehyde-type analogs has been reported [9], although the gene catalyzing the reverse reductive reaction has not yet been identified.

*Neurospora crassa* produces the structurally related salicylaldehyde sordarial (Figure 3). The biosynthetic mechanism of sordarial has been proposed [29]. In sordarial biosynthesis, it has been predicted that an aldehyde-type intermediate is released from PKS (SrdA) and cyclized by SrdC/D/E. The aldehyde-type intermediate is predicted to be modified by SrbB and SrdG to yield sordarial. In this pathway, an alcohol-type intermediate is thought to be produced from an aldehyde congener by an endogenous reductase; however, the gene responsible for the reduction of the aldehyde moiety has not yet been identified. SrdI, a homolog of MGG_10961/MoC19OXR1, is predicted to be involved in the oxidation of this alcohol-type intermediate [29]. The biological functions of sordarial are not known.

The extract of the PKS gene knockout strain fails to induce phytotoxic lesions on rice leaves, indicating that pyriculols are the sole lesion-inducing compounds produced by the wild-type strain under the culture condition used [28]. Interestingly, the PKS gene knockout strain is as pathogenic as the wild-type strain, demonstrating that pyriculols are not required for infection [28]. Further research is required to elucidate the biological roles of pyriculols.

## 4. Nectriapyrones

Nectriapyrone (Figure 1) is known as a polyketide compound produced by various fungi [30,31,32,33,34,35,36,37,38,39,40,41,42]. Interestingly, producers are mainly symbiotic fungi, including endophytes [34,36,37,38,41,43], plant pathogens [32,35], and sponge-associated fungi [30,31,33]. We recently found that nectriapyrone production can be induced in the rice blast fungus *P. oryzae* by disturbing the two-component signal transduction system [44]. We identified the nectriapyrone biosynthetic gene cluster and analyzed its physiological function.

Secondary metabolite production may be strictly regulated to produce under specific environmental conditions. Thus, we predicted that secondary metabolite production may be activated by disturbing signal transduction pathways involved in environmental responses. A two-component system (TCS) is a signal transduction system that regulates various cellular functions in response to environmental signals and is found in bacteria, archaea, plants, slime molds, and fungi [45,46]. The *P. oryzae* TCS was disturbed by disrupting *OSM1* and *PoYPD1*, encoding a HOG MAP kinase and a His-containing phosphotransfer (HPt) protein, respectively. This genetic modification induced the production of two polyketide compounds, nectriapyrone and its hydroxylated analog.

**Figure 4:**
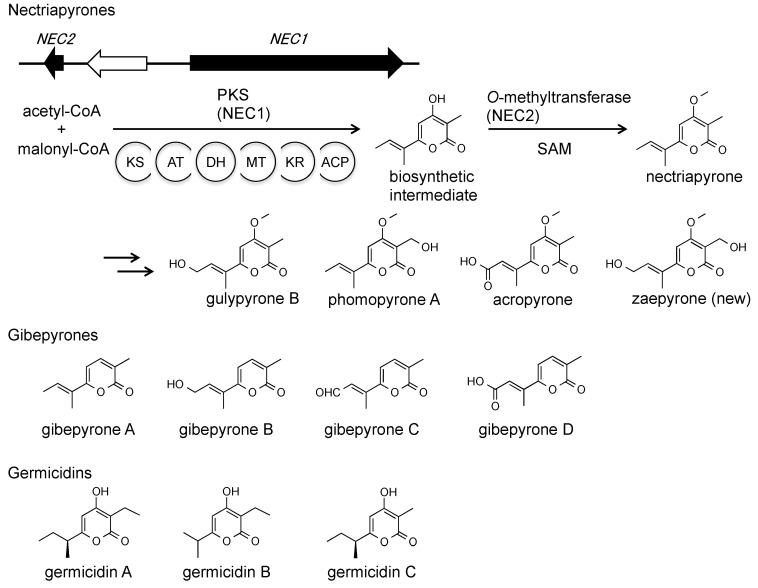
Nectriapyrones and related compounds.

We identified the nectriapyrone biosynthetic gene cluster consisting of a PKS gene (*NEC1*/*MGG_00806*) and an *O*-methyltransferase gene (*NEC2*/*MGG_14657*) (Figure 4). Overexpression of the two genes caused overproduction of nectriapyrone and five nectriapyrone analogs, including a new derivative, zaepyrone (Figure 4) [47,48,49,50]. Nectriapyrone shows similarity to the gibepyrones (Figure 4) from *Fusarium* spp. Gebepyrones do not have a methoxy group, and the *O*-methyltransferase gene is absent from the gibepyrone biosynthetic gene cluster [51]. Nectriapyrone also shows similarity to germicidins [52,53,54] from *Streptomyces* spp. (Figure 4). A type III PKS biosynthesizes the germicidin backbone [55]. In contrast, a type I PKS (NEC1) biosynthesizes the nectriapyrone backbone.

Nectriapyrones belong to the class α-pyrone. α-Pyrones have a wide range of biological activities [56,57]. For example, photopyrones are bacterial signaling molecules that control cell clumping [58]. Germicidins, produced by some *Streptomyces* strains, act as autoregulators of spore germination [52,53]. Some biological activities of nectriapyrone have been reported, although the functions of nectriapyrone in its producers are unknown. Nectriapyrone is toxic to bacteria, tumor cells, and plants [35,36,42,59]. It stimulates the formation of DOPA melanin in B16-F1 melanoma cells [39]. Nectriapyrone also inhibits monoamine oxidase in the mouse brain [60].

Identification of the nectriapyrone biosynthetic gene cluster allowed us to analyze the biological functions of nectriapyrones in the fungi that produce them [44]. While many nectriapyrone producers have been identified from plant pathogens, our data have indicated that nectriapyrones are not involved in rice infection and have different functions. The structure of nectriapyrone is similar to that of the germicidins produced by *Streptomyces* spp. Our data have indicated that nectriapyrone can control growth and pigment formation in *S. griseus* and has a growth-promoting effect on *P. oryzae* in interactions with *S. griseus*. Therefore, nectriapyrones may be involved in microbe-microbe interactions with other environmental organisms, including bacteria such as endophytic *Streptomyces* strains. To identify the active nectriapyrone analogs in this interaction, we analyzed the bioactivity of each analog (Figure 4) and found that nectriapyrone was the active analog, suggesting that other nectriapyrone analogs may be inactivated (detoxified) compounds of nectriapyrone.

## 5. Tenuazonic Acid

Tenuazonic acid (TeA, Figure 1), a tetramic acid derivative, is a well-known mycotoxin first isolated from the culture broth of *Alternaria tenius* in 1957 [61]. *Alternaria*, a ubiquitous plant pathogenic fungus, causes spoilage of various fruits and food crops in the field and post-harvest decay [62]. TeA has been detected in various *Alternaria*-contaminated vegetables, fruits, and crops [63,64,65]. The plant pathogenic fungi *P. oryzae* and *Phoma sorghina* (sorghum pathogen) are known as TeA producers [66,67]. *P. oryzae* also produces a related tetramic acid derivative from valine as a very minor product [68]. Among the *Alternaria* toxins, TeA is the most toxic and shows acute toxicity to mammals. The oral median lethal doses for male and female mice are 182 or 225 mg kg^−1^ and 81 mg kg^−1^ body weight, respectively [69,70]. The European Food Safety Authority evaluates TeA toxicity and determines its threshold of toxicological concern to be 1500 ng kg^−1^ body weight day [71]. TeA inhibits protein biosynthesis by inhibiting the release of the polypeptide from the ribosome [72]. TeA also has antitumor, antiviral, antibacterial, phytotoxic, and plant disease-controlling activities [69,73,74,75]. TeA has been shown to inhibit photosynthesis [76,77,78], and the potential use of TeA as a herbicide targeting photosystem II (PSII) has been proposed [79]. A recent paper [80] has shown that TeA inhibits plant plasma membrane (PM) H^+^-ATPase at micromolar concentrations. Inhibition of the PM H^+^-ATPase results in depolarization of the membrane potential and eventually necrosis. However, it is still unclear whether TeA is required for plant infection or not. We previously induced TeA production in *P. oryzae* and identified the biosynthetic gene *TAS1*, encoding the first reported fungal NRPS-PKS hybrid enzyme [81,82]. We also revealed the biosynthetic and induction mechanisms of TeA [83,84]. In this section, we review the data on the induction and biosynthesis of TeA.

The major fungal secondary metabolites, polyketides and nonribosomal peptides, are biosynthesized by PKSs and NRPSs, respectively. Fungal PKSs can be classified into three types. The first type is iterative type I PKSs, which consist of multiple catalytic domains, including ketosynthase (KS), acyltransferase (AT), and acyl carrier protein (ACP) main domains, along with several optional β-keto modifying domains, such as β-ketoacyl reductase (KR), dehydratase (DH), and trans-acting enoyl reductase (ER) domains [85]. The second type is the type III PKSs, which consist of a homodimeric KS [86]. The third type is fungal PKS-NRPS hybrid enzymes, which consist of an iterative type I PKS followed by a single module NRPS. These enzymes are involved in producing a wide variety of structurally diverse polyketide-amino acid hybrid compounds [87,88,89,90,91]. In fungal PKS-NRPS, the PKS part consists of KS, AT, and ACP domains, along with several modifying domains such as KR, DH, and methyltransferase (MT) domains. The NRPS part consists of adenylation (A), peptidyl carrier protein (PCP), condensation (C), and terminal release or cyclization (R, reductase or DKC, Dieckmann cyclization) domains [90]. PKS-NRPS hybrid enzymes have also been observed in bacteria [92,93]. Furthermore, a different type of hybrid enzymes, NRPS–PKS hybrid enzymes (which begin with an NRPS module), are also known. However, this type of enzyme has only been found in bacteria [94,95,96,97,98,99].

**Figure 5:**
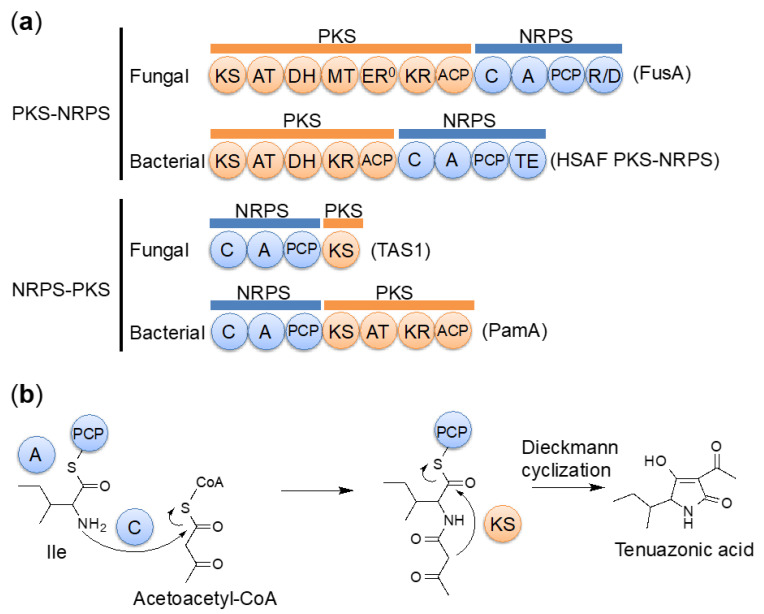
Tenuazonic acid (TeA) biosynthesis. (**a**) Comparison of domain structures. (**b**) Biosynthetic pathway of TeA.

TeA was shown to be a hybrid of an isoleucine and two acetates [100]. Because TeA has a tetramic acid-containing structure, TeA was also expected to be a product of a PKS-NRPS hybrid enzyme [101]. We successfully induced TeA production by disruption of the *OSM1* gene and 1% dimethyl sulfoxide (DMSO) treatment. OSM1 is the osmosensory mitogen-activated protein kinase (MAPK), which works downstream of the two-component signal transduction system involved in environmental responses. We identified the TeA biosynthetic gene *TAS1/MGG_07803* from the induced genes under inducing conditions [81,82]. TAS1 (tenuazonic acid synthetase 1) was not a PKS-NRPS hybrid enzyme but a NRPS-PKS hybrid enzyme. TAS1 is the first reported fungal NRPS-PKS hybrid enzyme consisting of an NRPS module of domains C-A-PCP and a terminal PKS KS domain (Figure 5a). TAS1 is a novel NRPS-PKS hybrid enzyme that starts with an NRPS module (C-A-PCP). This domain structure is very different from that of conventional fungal PKS-NRPS enzymes, which start with a PKS module (Figure 5a). The PKS portion of TAS1 has only a KS domain, in contrast to other NRPS-PKS hybrid enzymes. This KS domain is indispensable for TAS1 activity and has a unique sequence. By phylogenetic analysis, this KS domain was classified as an independent clade close to the type I PKS KS domain. We revealed that TAS1 synthesizes TeA from isoleucine and acetoacetyl-CoA (diketide) (Figure 5b). The unique KS domain catalyzes the final Dieckmann cyclization step for tetramic acid ring formation and TeA release, although involvement in diketide biosynthesis has been previously predicted [100]. In other NRPSs, bacterial NRPSs use the terminal thioesterase (TE) domain to catalyze substrate cyclization [102], whereas fungal NRPSs use the terminal condensation-like domain for substrate cyclization [103]. In addition, fungal PKS–NRPSs use the terminal reductase-like cyclization (DKC) domain for substrate cyclization [90]. These data indicate that TAS1 is a unique type of biosynthetic enzyme and may be used for the production of various compounds.

The KS domains of PKS normally catalyze the decarboxylative Claisen condensation of acyl and malonyl blocks to extend the polyketide chain [104]. In contrast, the terminal KS domain in TAS1 from *P. oryzae* conducts substrate cyclization [81]. Nonconventional KS domains with noncanonical roles have also been reported in type I PKS systems. A KS domain, one His residue of the catalytic triad is mutated, has been reported to only catalyze substrate transfer to the next domain in FR901464 biosynthesis [105]. KS3 of RhiE is required for vinylogous chain branching without polyketide chain elongation in rhizoxin biosynthesis [106]. Furthermore, homodimers of KS domains catalyze polyketide extension and substrate cyclization in a single catalytic pocket in type III PKSs [107]. However, a KS domain without a polyketide chain extension role has only been reported in TAS1. We analyzed the unique features of the TAS1 KS domain [84]. We found that the TAS1 KS domain is uniquely monomeric like NRPSs [108] although KSs are usually dimeric [109,110,111]. The 1.68 Å resolution crystal structure suggested that the substrate cyclization is triggered by proton abstraction from the active methylene moiety in the substrate by the catalytic H322 residue. We also found that TAS1 KS shows broad substrate specificity and promiscuously accepts aminoacyl substrates. Furthermore, this promiscuity could be increased by a single amino acid substitution in the substrate-binding pocket. These data provide hints to the substrate cyclization mechanism performed by the KS domain in TeA biosynthesis. These data also provide insight into how the NRPS-PKS hybrid enzyme accepts bulky amino acid-containing substrates.

**Figure 6:**
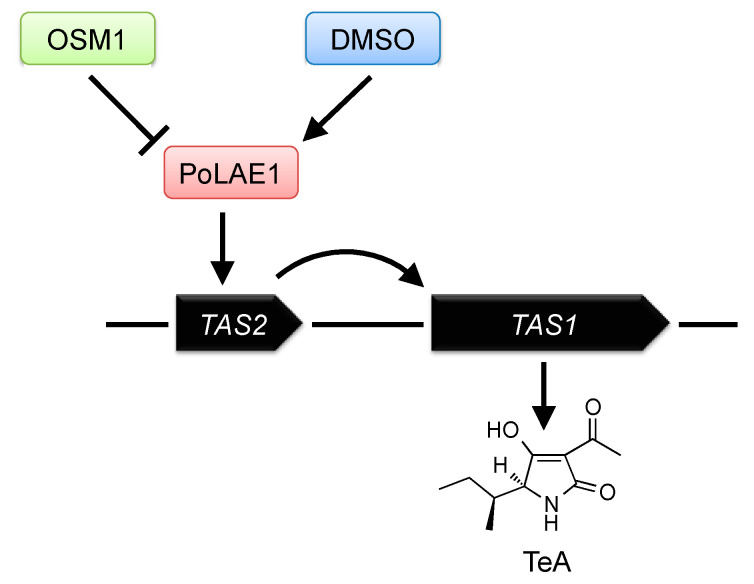
Regulation of TeA production in *P. oryzae*.

Transcription of secondary metabolism genes should be properly regulated in fungi in response to various environmental signals. Elucidation of the regulatory mechanism of these secondary metabolism genes is important for understanding the interaction between fungi and their environments. For example, elucidation of the regulatory mechanism of mycotoxin biosynthesis is important for protecting human and animal health by controlling mycotoxin production. As shown previously, TeA is biosynthesized in *P. oryzae* by TAS1, and its production is induced by osmo-sensory MAPK gene (*OSM1*) deletion or 1% DMSO treatment. However, the detailed regulatory mechanisms of TeA production have been unknown. We found two positive regulators of TeA production (Figure 6) [83]. In most cases, fungal secondary metabolites are produced using biosynthetic gene clusters. Many gene clusters have a gene for a cluster-specific DNA binding binuclear Zn(II)_2_Cys_6_-type transcription factor, which is known to be unique to fungi and activates the transcription of the clustered genes to produce a secondary metabolite [112]. These transcription factor genes include *Aspergillus nidulans aflR* for aflatoxin biosynthesis [113], *A. fumigatus gliZ* for gliotoxin biosynthesis [114], *Monascus purpureus ctnA* for citrinin biosynthesis [115], and *Fusarium sporotrichioides tri6* for trichothecene biosynthesis [116]. We identified a Zn(II)_2_Cys_6_-type transcription factor, TAS2 (MGG_07800), which regulates TeA production. *TAS2* is located in the upstream region of *TAS1* (Figure 6). In fungi, secondary metabolite production is also regulated by upper-level regulators rather than by cluster-specific transcription factors. These upper-level regulators, called global regulators, are trans-acting positive or negative transcriptional factors of secondary metabolite gene clusters. LaeA (loss of *aflR* expression) is a well-known global regulator of secondary metabolism identified and characterized in *Aspergillus* spp. [117,118,119,120]. Orthologs of LaeA have been identified in other fungi, including *Monascus pilosus*, *Cochliobolus heterostrophus*, and *Fusarium* spp. [121]. We identified a LaeA ortholog, PoLAE1 (MGG_01233), from *P. oryzae*. Analysis of *PoLAE1* deletion and overexpression strains showed that PoLAE1 positively regulated TeA production. We also revealed that two TeA-inducing signals, *OSM1* deletion and 1% DMSO treatment, were transmitted via PoLAE1. These results indicated that TeA production was regulated by two specific regulators, TAS2 and PoLAE1, in *P. oryzae* (Figure 6). Recently, it has been shown that TeA production is also induced by mycovirus infection via upregulation of *TAS2* [122].

## 6. Other Secondary Metabolites

*ACE1* (*MGG_12447*) is an avirulence gene, and isolates of *P. oryzae* carrying the *ACE1* gene are specifically recognized by rice cultivars carrying the resistance gene *Pi33* [87]. This recognition activates defense responses in resistant plants. *ACE1* is a secondary metabolism gene and encodes a PKS-NRPS hybrid enzyme. The secondary metabolite whose synthesis is dependent on ACE1 is predicted to be recognized by resistant rice plants. *ACE1* is located in an infection-specific gene cluster consisting of 15 genes [7]. Fourteen of the 15 genes are predicted to be involved in secondary metabolism as they code for proteins such as a second PKS-NRPS (SYN2), two enoyl reductases (RAP1 and RAP2), and a putative Zn(II)_2_Cys_6_-type transcription factor (BC2). The *ACE1* gene cluster shows a rice-infection-specific expression pattern. Heterologous co-expression of *ACE1* and *RAP1* in *Aspergillus oryzae* causes production of an amide compound similar to the PKS-NRPS-derived backbone of cytochalasans [123]. Bioactivity analysis shows that the produced compound is not responsible for the observed *ACE1*-mediated avirulence. These data suggest that the active final product may be a cytochalasin-like compound.

Penicillin production in rice blast fungus has been recently shown [124]. Overexpression of a *laeA* homolog (*MoLAEA*/*MGG_07964*) increases the production of penicillin G compared to the wild type strain. In contrast, the silenced strain does not produce penicillin G. The putative NRPS gene (*MGG_14767*) for penicillin G biosynthesis is 3-fold upregulated in the overexpression strain, whereas it is 3.8-fold downregulated in the knockdown strain. Transcriptomic data show that MoLAEA regulates genes involved in secondary metabolism. This *laeA* homolog (*MoLAEA*/*MGG_07964*) is different from the *laeA* homolog (*PoLAE1*/*MGG_01233*) involved in TeA production. Multiple *laeA* homologs may be involved in the control of secondary metabolite production.

*ABM* (*MGG_04777*) is a monooxidase gene located in a putative secondary metabolite biosynthetic gene cluster containing a polyketide synthase gene (*MGG_04775*) [125]. While the role of Abm (antibiotic biosynthesis monooxygenase) in this gene cluster is unknown, Abm can convert endogenous free jasmonic acid (JA) into 12OH-JA in *P. oryzae*. Such fungal 12OH-JA is secreted during infection and helps evade the defense response by inhibiting the induction of JA signaling. In contrast, loss of Abm in *P. oryzae* causes accumulation of methyl JA (MeJA), which induces host defense and blocks fungal infection. Furthermore, Abm itself is secreted after infection and is predicted to convert plant JA into 12OH-JA to help host colonization. *P. oryzae* also produces other plant hormones, abscisic acid (ABA) [126], cytokinins (CKs) [127], and auxins [indole-3-acetic acid (IAA)] [128], and the biosynthetic genes for ABA and CKs have been identified and characterized [129,130]. Gene knockout of *MoABA4*/*MGG_07514*, homologous to the *Botrytis cinerea ABA4* gene responsible for ABA biosynthesis, reduces ABA levels by two-fold [129]. The virulence of the Δ*Moaba4* mutant is strongly compromised, suggesting that ABA contributes to the virulence of this fungus. *CKS1*/*MGG_04857* encodes a putative tRNA-Isopentenyl transferase (tRNA-IPT) protein essential for CK biosynthesis [130]. The interaction between rice and the Δ*csk1* strain has been characterized. This analysis has shown that *P. oryzae*-derived CKs are required for full virulence by affecting rice defenses, nutrient distribution, and fungal oxidative stress tolerance.

## 7. Conclusions

*P. oryzae* is rich in secondary metabolism genes, and some of the secondary metabolites are expected to be involved in rice infection. Here, this review focused on the biosynthesis and biological roles of secondary metabolites in *P. oryzae*. Five groups of secondary metabolites (melanin, pyriculols, nectriapyrones, TeA, and penicillin G) have been shown to be produced by *P. oryzae*. Biosynthetic genes for three (nectryapyrone, TeA, and penicillin G) of the five groups of secondary metabolites have been identified by activating secondary metabolism. Nectryapyrones and TeA were induced by manipulating the factors involved in the TCS. Penicillin G was induced by overexpression of a *laeA* homolog. Activation of secondary metabolism is a useful method for the identification of secondary metabolites. Melanin is a well-characterized secondary metabolite that is essential for rice infection. Identification and gene manipulation of the biosynthetic genes revealed that three groups of secondary metabolites (pyriculols, nectriapyrones, and TeA) are not required for rice infection. Nectriapyrones and penicillin G show antibacterial activity and are predicted to be involved in interactions between bacteria. Controlling plant pathogenic fungi is important in agriculture. Unveiling the roles of secondary metabolites of plant pathogens will help in developing agrochemicals.

## Abbreviations

PKS	Polyketide synthase
NRPS	Nonribosomal peptide synthetase
1,8-DHN	Dihydroxynaphthalene
1,3,6,8-THN	1,3,6,8-Tetrahydroxynaphthalene
1,3,8-THN	1,3,8-Trihydroxynaphthalene
MBIs	Melanin biosynthesis inhibitors
TCS	Two-component system
HPt	His-containing phosphotransfer
DOPA	3,4-Dihydroxyphenylalanine
TeA	Tenuazonic acid
PSII	Photosystem II
PM	Plasma membrane
KS	Ketosynthase
AT	Acyltransferase
ACP	Acyl carrier protein
KR	β-Ketoacyl reductase
DH	Dehydratase
ER	Enoyl reductase
MT	Methyltransferase
A	Adenylation
PCP	Peptidyl carrier protein
C	Condensation
R	Reductase
DKC	Dieckmann cyclization
DMSO	Dimethylsulfoxide
MAPK	Mitogen-activated protein kinase
TE	Thioesterase
JA	Jasmonic acid
MeJA	Methyl jasmonic acid
ABA	abscisic acid
CKs	cytokinins
IAA	indole-3-acetic acid
tRNA-IPT	tRNA-Isopentenyl transferase
